# *Caenorhabditis elegans*, a Host to Investigate the Probiotic Properties of Beneficial Microorganisms

**DOI:** 10.3389/fnut.2020.00135

**Published:** 2020-08-21

**Authors:** Cyril Poupet, Christophe Chassard, Adrien Nivoliez, Stéphanie Bornes

**Affiliations:** ^1^Université Clermont Auvergne, INRAE, VetAgro Sup, UMRF, Aurillac, France; ^2^Biose Industrie, Aurillac, France

**Keywords:** *Caenorhabditis elegans*, probiotics, lifespan, pathogens, stress response, immunity

## Abstract

*Caenorhabditis elegans*, a non-parasitic nematode emerges as a relevant and powerful candidate as an *in vivo* model for microorganisms-microorganisms and microorganisms-host interactions studies. Experiments have demonstrated the probiotic potential of bacteria since they can provide to the worm a longer lifespan, an increased resistance to pathogens and to oxidative or heat stresses. Probiotics are used to prevent or treat microbiota dysbiosis and associated pathologies but the molecular mechanisms underlying their capacities are still unknown. Beyond safety and healthy aspects of probiotics, *C. elegans* represents a powerful way to design large-scale studies to explore transkingdom interactions and to solve questioning about the molecular aspect of these interactions. Future challenges and opportunities would be to validate *C. elegans* as an *in vivo* tool for high-throughput screening of microorganisms for their potential probiotic use on human health and to enlarge the panels of microorganisms studied as well as the human diseases investigated.

## Introduction

With the advent of probiotic microorganisms, including lactic acid producing bacteria (LAB), in the treatment of diseases related to dysbiosis, there is the question of understanding the mechanisms of action of these strains. How to efficiently screen microorganisms' collections to select new probiotics for innovative therapeutics applications is an emerging question as well.

Probiotics have been officially defined in 2001 by both the Food and Agriculture Organization of the United Nations (FAO) and the World Health Organization (WHO) as “live microorganisms which, when administered in adequate amounts, confer a health benefit on the host” (1). A new name is increasingly used to replace the term probiotic: live biotherapeutic products (LBP). These LBP are biological products containing live biotherapeutic microorganisms (LBM) used to prevent, treat, or cure a disease or condition of human beings, excluding vaccines (2). Under this appellation, a wide variety of microbial species is found within both prokaryotes and eukaryotes (yeasts) although there are mainly lactic bacteria such as *Lactobacillus* strains.

In general, the mechanisms of action of probiotic species are poorly characterized and often based on empirical data as survival studies in the gut or immunomodulation (3, 4). At present, selection of new probiotic strains is based on a series of tests both *in vitro* and *in vivo*. The main *in vitro* tests commonly used are: the tolerance to acidity and gastric enzymes, the tolerance to bile, and intestinal enzymes, the adhesion to intestinal cells and the persistence in the digestive tract, and the lack of pathogenic behavior (5). *In vitro* tests are informative but complementary *in vivo* approaches is mandatory for a better characterization of probiotics mechanism of action but also safety aspects.

The selection of new microbial strains with probiotic potential as well as the characterization of their properties have been based on experiments using *in vitro* models (cell cultures). These have an undeniable advantage: strict control of the experimental conditions (e.g., nature of the culture media, quantity of microorganisms, temperature, pH, etc.) as well as interactions between a limited number of microorganisms, most often using binary models. This facilitates the analysis and interpretation of the observed effects. However, the experimental conditions are particularly distant from the complex conditions found in a host. In addition, research into probiotic microorganisms is facing new challenges with increasing regulatory pressure from the European Union and the United States of America. These regulatory obstacles impose on pharmaceutical laboratories manufacturing probiotics and therefore on research teams, the use of relevant methods and models to study probiotic strains, by combining *in vitro* and *in vivo* approaches. It is only with this finer characterization that the laboratories will be able to justify more firmly the interest of using their products for health applications (6). However, in the same way as for *in vitro* models, depending on the scientific (historical) context and the objective to be achieved, it will be advisable to select the most pertinent model before the final essential study which is to carry out clinical trials in humans (7). Regarding to *in vivo* tests, many studies involve laboratory animals (e.g., rats, mice, rabbits, or guinea pigs) or clinical trials on the human population for the last stages of research. These clinical trials, although very important to determine the effectiveness of the strain and the potential harmful effects, are particularly expensive, time-consuming and difficult to implement (8). There is a need for *in vivo* alternatives, easier to handle but sufficiently close to humans for the *in vivo* and large-scale probiotic studies. In any case, the thorough study of gut microbiota remains difficult and requires a lot of steps combining *in vitro* studies, tests on laboratory animals and finally on humans (9).

The current number of *in vivo* methods for fast and reliable analysis of collections using complex multicellular models is too limited. Mainly for cost reasons, infrastructure and ethics, it is definitely not possible anymore to use quantities of laboratory animals such as mice or rats during the early stages of research (10, 11). For over 30 years, at least 4,000 researchers around the world have turned to *Caenorhabditis elegans* in fields such as ecotoxicology, neurobiology, degenerative disease, aging, host-microorganism interactions, and recently virus-host interactions (12–14). *C. elegans*, which was described for the first time in 1900 by Maupass (15), was introduced into laboratories in the late 1960s by Sidney Brenner who made it a model in genetics. His work, which won him the Nobel Prize in Medicine in 2002, allowed a detailed understanding of the molecular mechanisms of the regulation of gene expression, cell polarity, apoptosis, organogenesis, and aging (16, 17). *C. elegans* is a 1 mm long worm, living in the soil and usually feeding on bacteria. It is a self-fertilizing hermaphrodite nematode with many laboratory advantages. Among these advantages are the simple growth conditions, a short generation time (18), a transparent body facilitating fluorescence microscopy observation, the ability for genetic engineering and a low cost of use. Thanks to its sensory neurons allowing it to detect different chemical signatures, it is able to select its food and at the same time avoid pathogenic microorganisms (19). The generation of one million mutants obtained in a few weeks will be contained in 20 Petri dishes at a cost equivalent to that of a single mouse (20). Considering the morphological aspect of the animal, its intestine is a major organ with one third of the total somatic mass (21). The morphology of intestinal cells as well as the phenomena of endocytosis and exocytosis display similarities with humans (22). The 97 Mbp worm's genome is fully sequenced (23) and contains 19,099 genes, almost as much as the human genome. Seventy-four percent of human proteins have homologs in the model organism *C. elegans* (24). This sequence and annotation knowledge allowed the generation by the *Caenorhabditis* Genetic Center (CGC) of more than 12,000 different strains (wild, mutants), all genetically characterized, facilitating the mechanistic research using the nematode. Key genes regulating in a global way the metabolism and the life expectancy of *C. elegans* such as the *daf-2* and *daf-16* genes, respectively encoding for an insulin receptor and for a Forkhead box O (FOXO) transcription factor, were identified. They are involved in the expression of genes encoding chaperone, antioxidant, or antimicrobial proteins via the Insulin/IGF-1 mediated signaling pathway (IIS), all leading to the modulation of the worm's health condition and consequently of its lifetime (25–27).

Several antimicrobial pathways found in mammals are also found in the worms suggesting it is a powerful model to explore immunological mechanisms (28, 29). Finally, since the first infection model involving *Pseudomonas aeruginosa* in 1999 (30, 31), a lot of studies reported the susceptibility of the worm to numerous bacterial and fungal pathogens, some of them infecting humans such as *Pseudomonas aeruginosa* (32), *Enterococcus faecalis* (33), *Staphylococcus aureus, Salmonella, Escherichia coli* O157:H7, or *Klebsiella pneumoniae* (34–37). Considering all these points, *C. elegans* could be a relevant go-between host model between *in vitro* and mammalian models for the investigation of host-microorganism interactions at the molecular level using direct genetic and mechanistic approaches.

This review is focused on studies using *C. elegans* to investigate its interaction with lactic acid bacteria having evidenced probiotic capacities. The pertinence and the advantages of using *C. elegans* as a model organism to identify new potentially probiotic strains are analyzed. The new way to use the worm, with the aim to screen microorganisms' collections for specific probiotic characters, is also detailed in the manuscript. This manuscript was written from an exhaustive review of the scientific literature related to the *C. elegans* model and the *in vivo* characterization of microbial strain with probiotic potential. All recent articles have been considered without distinction and studied objectively to avoid bias. The compilation of bibliographic data, from the PubMed database, was carried out in three stages. The first, relating to the comparison of experimental models, was done using the keywords experimental models, *in vivo*, comparison, mice, rat, zebrafish, *C. elegans, Drosophila melanogaster*, and *Galleria mellonella*. The second, in connection with the use of the *C. elegans* model as the study of probiotics, required the following key words *C. elegans*, probiotic, live biotherapeutic microorganism, mechanisms, immunity, and pathway. Finally, for the third step, the keywords *C. elegans*, high-throughput, translational, and prospects were used. In all three steps, the logical operator “AND” was used.

Fifty-two publications focused on *C. elegans* and probiotics are referenced in PubMed database, 19 out of 48 have been published from 2018, 11 of whom in 2019 and already 11 in 2020, confirming the emergence of this model in this research domain.

## *C. elegans* Model Advantages and Limitations Compared to Other *in vivo* Models

The use of *in vivo* techniques to explore the world of probiotics has made it possible to describe a large variety of models with very varied complexities. These can be as simple as multicellular organisms like worms, flies (invertebrates) as well as being particularly sophisticated like knockout mice or humans (clinical trials). All these models provide the scientific community with a very large amount of information, but they also have certain drawbacks. Consequently, it is essential to choose with discernment and rigor the model which will be the most adapted to the scientific question based on technical, ethical, economic aspects, and according to scientific context. Therefore, while the characterization and evaluation of the properties of a probiotic strain should be carried out directly within the target population, a preselection of the strains is essential before testing it using appropriate *in vivo* models (6, 38). Two kinds of models are commonly used, the vertebrate and the invertebrate organisms. The Table 1 lists the advantages and the limitations of the most commonly used models in the context of probiotics characterization but among the limits of their use are ethics and legislation.

**Table 1:** Comparison of vertebrate and invertebrate models used for microorganism—host, microorganism—microorganism and microorganism—microorganism—host interactions.

	**Vertebrates models**			**Invertebrates models**		
	***Mus musculus***	***Rattus norvegicus***	***Danio rerio***	***Drosophila melanogaster***	***Galleria mellonella***	***Caenorhabditis elegans***
Full genome sequence available	Yes	Yes	Yes	Yes	Yes	Yes
Resources needed in the laboratory	Very high	Very high	High	Low	Low	Low to high
Ease of implementation	High	High	Low	Low	Low	Low
Mutants available in public bank	Yes	No	Yes	Yes	No	Yes
RNAi bank available	No	No	No	Yes	No	Yes
Major intestinal taxa	*Firmicutes, Bacteroidetes, Actinobacteria*	*Firmicutes, Bacteroidetes, Proteobacteria*	*Proteobacteria, Fusobacteria*	*Lactobacillus, Acetobacter*	No microbiota in laboraroty condition	No microbiota in laboratory condition
Survival at 37°C (human body temperature)	Yes	Yes	No	No	Yes	No
Immunity	Innate and adaptative	Innate and adaptative	Innate and adaptative	Innate	Innate	Innate
Axenic animals	Yes but technically demanding and very expensive	Yes but technically demanding and very expensive	Yes but limited in time	Yes	Yes	Yes
Compatibility with human origin microorganisms	Variable	Variable	Yes but limited to aerobic taxa	Yes but limited to aerobic taxa	Yes but limited to aerobic taxa	Yes
Screening platform	No	No	Yes	Yes	Yes	Yes
Usable for high-throughput studies	No	No	Yes	Variable	Yes	Yes
Microscopic observation on live whole animal	No	No	Yes	No	No	Yes
Ethical issues	Yes	Yes	No	No	No	No
Other limitations	Impact of skin, fur, digestive tract and behavior on microbial communities	Impact of skin, fur, digestive tract and behavior on microbial communitiesLack of mutants	High variability of the gut microbiota based on experimental and environmental conditions	Low diversity of the gut microbiotaHigh-throughput methods difficult to implementInjecting a pathogen bypasses the early stages of infection	No mutants Low standardization between laboratories	Time consuming without automationImpact of temperature on microbial metabolism

*Adapted from Douglas (7) and Nathan (39)*.

### Vertebrate Models

The scientific community now has vertebrate animal models allowing investigations in most fields of biology. The best known are the rat (*Rattus norvegicus*), the mouse (*Mus musculus*), the zebrafish (*Danio rerio*), the pig (*Sus domesticus*), the guinea pig (*Cavia porcellus*), the macaque (*Macaca mulatta*), the cat (*Felis sylvestris catus*), or the dog (*Canis lupus familiaris*) to name a few. However, among this multitude and due to their similarities to humans, only a few models are commonly used in the laboratory, mainly due to regulatory and ethical constraints, the complexity of use, but also the particularly high cost they can represent. The pig (*Sus domesticus*) is notably used for the selection of probiotics, often directed against infections with *Escherichia coli* or *Clostridioides difficile*, but rarely for mechanistic purposes. The use of the dog or the monkey is quite marginal and is mainly restricted to very specific areas because their implementation requires considering considerable ethical considerations (6).

#### The Mouse *Mus Musculus*

The laboratory mouse *M. musculus* is a powerful model for the evaluation of host-microbiota interactions, in relation to human biology (40–42). About 99% of mouse genes are shared with humans, and the two organisms share significant similarities in the microbiome (*Firmicutes, Bacteroidetes*, and *Actinobacteria*). Many genetic tools are available to the scientific community: many strains inbred or not, a collection of mutants and the possibility of carrying out RNA interference *in vivo*. However, experimental limitations exist. The skin, fur, and oropharyngeal structures, compartmentalization of the digestive tract and behavior are different from those of humans and can have a great influence on microbial communities and therefore their impact on the host (43). A significant proportion of microorganisms from human origin are unable to colonize the murine intestine and the taxa which colonize it do not induce certain responses usually observed in the presence of the native microbiota (44, 45).

#### The Rat *Rattus Norvegicus*

The rat is the second rodent species used in research as a model for preclinical study (46). Closer physiologically to humans, it allows to obtain experimental results that are more easily transposable to humans (47). Like for the human, the intestinal microbiota of the rat is dominated by three large phyla: *Firmicutes, Bacteroidetes*, and *Proteobacteria* (48). The animal is used to study the impact of bacteria with probiotic potential on the intestinal level, in the case of inflammatory syndromes, in particular in connection with obesity (49–52). However, the development of the rat model has until recently been slowed down by the lack of available mutants, the first of which were only generated by genome editing tools in 2010 (53). Since then, different methods of transgenesis have been successfully applied which should accelerate the development of mutants (54).

#### The Zebrafish *Danio Rerio*

The zebrafish *D. rerio*, with its varied microbiota, is becoming a powerful model for studying the complexity of host-microbiota interactions. It has several characteristics that make it an attractive experimental system. The external fertilization and transparency of embryos and larvae allow the visualization of developing cells as well as the colonization of microorganisms. Its rapid development, its small size, its high degree of homology with mammals make the zebrafish a tool interesting for mechanistic studies, not only in the adaptive immune system (TLR and NOD receptors), but also in the digestive system (43). Limitations of the zebrafish model in microbiota research include differences in environmental conditions compared to humans and other model organisms (55). Although the fecal microbiota of humans and rodents can vary, it is still dominated by the phyla *Firmicutes* and *Bacteroidetes*. The zebrafish gut microbiota is dominated by *Proteobacteria* and *Fusobacteria*, with variable presence of *Firmicutes, Cyanobacteria*, and *Actinobacteria*, and it varies considerably from one laboratory to another, notably depending on the diet (56, 57).

#### Ethics and Legislation

Researchers who need animal models are faced with ethical and legal considerations. In addition, public opinion is often not aware of the economic and societal importance of the proposed research or of the regulatory context, which also limits the unethical use of animal suffering. Researchers must also actively ensure that animal models (i) are scientifically (and statistically) validated (ii) cannot be replaced by *in vitro* alternatives and (iii) minimize animal suffering by limiting the number of animals and the duration of the experiment to what is statistically required. Research strategies and methods must be constantly challenged and objectively examined against the 3R rule established more than 50 years ago, that is, using the possibilities of replacement, reduction, and refinement (58). In addition to the proper management of pain by analgesia and anesthesia, the welfare facilities have improved considerably in accordance with the latest American and European guidelines for the accommodation of animals. Mice and rats must have sufficient space, of sufficient complexity, to express a wide range of normal behaviors and to provide enrichment opportunities to promote physical exercise, foraging, eating, manipulation and cognitive activities (6, 59).

### Invertebrate Models

Among the invertebrate laboratory models are the fly (*Drosophila melanogaster*), the greater wax moth (*Galleria mellonella*), and the roundworm (*Caenorhabditis elegans*). These models are commonly used in the laboratory, mainly due to the facilities in terms of legislation and ethics, their easy way of use, but also their low cost. The potential probiotic capacities of new strains need the use of different and complementary approaches to be elucidated. The invertebrates represent in-between models complementary to *in vitro* approaches and allowing the identification of scientific hypothesis justifying the use of vertebrate models.

#### The Fly *Drosophila Melanogaster*

*D. melanogaster* can allow reliable validation of probiotic effects on a living organism: high throughput screening capabilities, inexpensive and rapid reproduction, and microbiome easy to handle. In addition, many tools for studying host-microbe interaction in *D. melanogaster* are already available due to its rich history in pathogen research (60). Compared to the gastrointestinal tract of mammals, the intestine of *D. melanogaster* has several major differences, but the gastrointestinal physiology, anatomy and signaling are highly conserved (61). A wide range of strains of *D. melanogaster* available from public banks can be derived germ-free and easily maintained without requiring expensive animal facilities, equipment, and technicians (62). Compared to the human microbiota, the *D. melanogaster* microbiota has a low microbial diversity (1 to 30 species) and is generally dominated by *Lactobacillus* and *Acetobacter* (63–65). These characteristics make *D. melanogaster* an ideal high-throughput *in vivo* model for understanding host-microbiota interactions (66), in particular for screening bacteria with probiotic potential (67). However, there are weaknesses limiting the use of the fly as a model. Indeed, the infection of the animal by a microbial strain is carried out by a direct injection, which bypasses the mechanisms put in place during the first stages of an infection.

#### The Greater Wax Moth *Galleria Mellonella*

While it is not (yet) as genetically modifiable as *D. melanogaster* or *C. elegans*, the simplicity of handling and infection of the larvae of *G. mellonella* combined with their survival at 25 and 37°C makes this animal a promising model (39). In research on bacterial (68, 69) and fungal (70, 71) pathogenesis, the larvae of *G. mellonella* have been shown to be a relevant model of infection. In their study, Vilela et al. used larvae as a model for pathogenic yeast infection *C. albicans* and co-infection with a beneficial strain of *Lactobacillus acidophilus* (72). Unlike *D. melanogaster* and *C. elegans*, fungal or bacterial infections and the immune defense mechanisms of the host in *G. mellonella* seem to be very close to those encountered in humans (39). Conversely, the lack of a complete genome sequence, the absence of mutant strains and the need for standardization between different sources and laboratories (39, 69), are currently curbing the enthusiasm for this model.

#### The Roundworm *C. elegans*

The strength of *C. elegans* as a model organism for microbiome research is its ability to conduct high- throughput experiments with a gnotobiotic organism and to explore the complex cause and effect relationship between presence and absence of a microbial species and a phenotype reflecting good or bad health. In fact, the microorganisms that pass through the pharynx to the intestine represent the only nutritive source for nematodes. They can thus influence the physiology of the animal through their metabolites (73). In addition, the use of nematodes for the screening of probiotics is favored by the possibility of easily monitoring anti-aging markers, as well as the storage of body fat (74, 75). In addition, several genes involved in the stress response and linked to immunity are highly conserved between humans and nematodes (76).

Today, the growing interest in the effects on longevity due to probiotics has led to the need for practical *in vivo* models to understand the mechanisms of probiotic activity. In recent years, the *C. elegans* nematode has become a powerful animal model for studying host-probiotic interactions. Its advantages include ease of handling, transparency of the body, short lifespan, and the absence of ethical issues. Another important tool of *C. elegans* is the availability of transgenic animals. Many mutants, available in libraries (CGC, https://cgc.umn.edu/), can help study the mechanism of action at the molecular level of a compound or pathways involved in the host-microorganism interaction. A collection of *E. coli* strains enabling RNAi feeding to be carried out in *C. elegans* is also available to the scientific community (*C. elegans* RNAi Collection, The Wellcome CRC Institute, University of Cambridge, UK, https://www.sourcebioscience.com/life-science-research/clones/rnai-resources/c-elegans-rnai-collection-ahringer/). For example, in an infectious context, the use of transgenic GFP nematodes makes it possible to study the response *in vivo* by fluorescence analysis (77, 78). Also, several online and freely accessible tools allow any type of researcher (beginner, confirmed, or expert) to access a large amount of data on the model, covering as well the fields of fundamental biology as applied (WormBase, https://wormbase.org/; WormWiring, https://www.wormwiring.org/; WormAtlas, https://www.wormatlas.org/; WormBook, http://wormbook.org/; OpenWorm, http://openworm.org/).

*C. elegans* model allows transcriptomic, proteomic and networks analysis, offering promising mechanistically insights opportunities (79–81). Moreover, *C. elegans* opens new perspectives in developing assays to investigate the cytotoxicity of new bioactive molecules or microbial pathogenicity and host defense mechanisms and gives new strategies to understand the mode of action (specific chemical, virulence factor). *C. elegans* allows the following of a microorganism in a whole animal with an integrated point of view, considering the gene expression using transcriptomic and proteomic approaches as well as metabolomics and physiology of the entire organism (82).

Because of its easy-to-use potential and because of the large panel of mutants (*sek-1* or *daf-16*::*gfp* mutants for example), *C. elegans* appears an efficient *in vivo* model that could be used for the screening of microorganisms collections with the aim to identify new potential probiotic strains and to investigate the molecular mechanisms involved. The potential probiotic capacities of new strains need the use of different and complementary approaches to be elucidated.

## *C. elegans*: Model Limitations and Practical Interpretation Pitfalls

Like all study models, the *C. elegans* nematode is not without limitations which could bias the interpretation of the results obtained (39). From a practical point of view, although the worm is rather easy to use, its total control requires a significant training. Some crucial experimental points can be quite tricky such as synchronization step or daily worms counting on solid medium spread with yeasts for example; in the absence of an automated system, its implementation is particularly time-consuming. The cell number of the worm is anyway limited (20 intestinal cells out of 959 cells for an entire worm) questioning about the relevance of the phenotypes observed and their extrapolation to humans which are composed of many different and complex tissues. In addition, the impossibility of maintaining it at 37°C is an important limit since it can greatly affect the metabolism of the microorganisms studied. This makes it tricky extrapolating the results to humans. Moreover, the main way to provide the microorganism of interest is to feed with, which represents a risk to see them destroyed by the digestive system of the animal. Because of the worm's ability to choose its food, it is essential to ensure that the microorganism has been consumed because otherwise, the results obtained will be distorted.

In the laboratory, the nematode is conventionally propagated on axenic cultures preventing any installation of a complex microbiota within its digestive tract, which causes a drift compared to wild physiological conditions. In addition, the absence of an adaptive immune system and a variable correlation of virulence factors with mammals limit the scope and extrapolation of the experimental results.

Nevertheless, an objective critical analysis of the advantages and disadvantages of the animal still makes it possible to demonstrate that this model is suitable for carrying out preclinical tests intended for a future application in human. Indeed, the nematode represents a good intermediary between *in vitro* cell cultures and mammals. Compared to cell cultures, *C. elegans* has the obvious advantage of being an entire organism with different cell and tissue structures, which the *in vitro* approach does not or hardly allow. Because of the absence of ethical problems and of a lower cost, it allows a more precise orientation of future experiments on mammals decreasing at the same time the number of animals to use and/or sacrifice.

## Probiotic Microorganisms Influence on *C. elegans* Lifespan

Safety assessment is a mandatory to characterize the innocuity of any probiotic stains. In this context, one of the *in vivo* strategy to be considered is the study of the survival of the nematode (83).

### Positive and Negative Effects of Probiotics on the Worm Lifespan

As most living organisms, the nutrient sources used for the growth and propagation of *C. elegans* have a direct impact on its health, its defenses against pathogens and its lifespan (84).

Studies showed that the worm lifespan is increased when it is propagated on axenic cultures (85), or on agar medium with UV-killed bacteria (86, 87). Moreover, *C. elegans* fed with some probiotics showed a lower bacterial load in its intestine than those fed with *E. coli* OP50, allowing an extension of the worm's lifespan (88). The behavior of *C. elegans* in contact with probiotic microorganisms, which are supposed to improve the health of the host, needed investigation.

**Figure 1:**
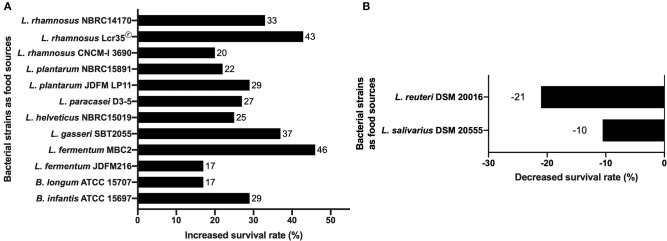
Effects of bacterial probiotic strains on *C. elegans* lifespan. **(A)** Lifespan of *C. elegans* N2 fed with lactic acid bacteria compared to worms fed with *E. coli* OP50. The increase in the survival rate is represented for each strain as a percentage of the survival rate of *C. elegans* fed on *E. coli* OP50 (11, 27, 34, 36, 76, 84, 89–91). **(B)** Negative effects of *L. salivarius* and *L. reuteri* on *C. elegans* lifespan. The decrease in the survival rate is represented for each strain as a percentage of the survival rate of *C. elegans* fed on *E. coli* OP50 (92). The decrease in the survival rate is represented for each strain as a percentage of the survival rate of *C. elegans* fed on *E. coli* OP50.

Focusing on Lactic Acid Bacteria (LAB), some lactobacilli strains significantly increase *C. elegans* N2 (wild type) mean lifespan compared to conditions where it is fed with the standard food *E. coli* OP50. Ten lactobacilli strains (*L. fermentum, L. gasseri, L. helveticus, L. paracasei, L. plantarum*, and *L. rhamnosus*) and two bifidobacteria strains (*B. infantis* and *B. longum*) (Figure 1A) have been shown to increase nematode survival of *C. elegans* wild-type N2 from 17% to a maximum of 46% (11, 34, 36, 76, 89–91). Other studies showed that *Lactobacillus* (*L. helveticus, L. plantarum*, and *L. rhamnosus*) or *Bifidobacterium* (*B. infantis* and *B. longum*) grown under anaerobic conditions, also allowed the increase of *C. elegans* longevity with respectively 25, 22, 33, 29 and 17% of augmentation (27, 84). It has been shown that the significant increase in *C. elegans* survival by *Lactobacillus gasseri* SBT2055 occurs with both live bacteria and UV-killed bacteria. This potentiation was correlated with the ratio *E. coli* OP50/*L. gasseri* LG2055 used, i.e., the smaller the ratio the larger the survival rate (86). A recent study has shown an increase in longevity and an improvement in the physical functions of the host when it is fed with heat-killed probiotic strains (*L. paracasei* D3-5 and *L. plantarum* SK-9). According to the authors, these properties are associated with an anti-aging effect by maintaining high muscle mass and normal body size (91). Another study showed that a mix of *B. infantis* and *E. coli* OP50 at different proportions influenced the longevity of the worm. This information shows that it is necessary to consider the quantity of microorganisms brought to the nematode, especially the proportions in the case of a mixture, to make rigorous experiment and obtain relevant data. Also, it should be noted that the food used routinely for the maintenance of worms can have an impact on the way the worm responds to a biotic or abiotic stress. From where, there is an interest to use *E. coli* OP50, known to have little effect in *C. elegans* and should be always used as a control condition during experiment. Considering all these studies, we noticed that LAB strains exert a positive effect on the worm lifespan either alive or dead or both. Further investigations are needed to understand why this point influence the beneficial effect of a strain. It could be a strategic data to include in the process of development of new LBP. The authors agreed to suggest that the mechanism used by the bacteria to promote the longevity of the worm involves the p38 MAPK (i.e., p38 Mitogen-Activated Protein Kinase) signaling pathway until the induction of the expression of skn-1 gene and the target genes of SKN-1, encoding the antioxidant proteins (phase 2 detoxification enzymes) (86). They have also found that the cell wall (lipoteichoic acids) of Bifidobacteria plays a key role in increasing the longevity of nematodes, which is in a good correlation with other works focused on *B. longum* (93, 94). Another study showed the stimulation of *C. elegans* longevity through *pmk-1* and nuclear hormone receptor (NHR), a receptor family associated with longevity in humans and animals, including *C. elegans*, even if the underlying mechanism in human remains unclear (95). Recently, the authors have shown that a mutation of the Toll-like receptor homolog TOL-1 in the nematode induced a significant increase in longevity of the host in the presence of *B. infantis*, but not in the presence of *Bacillus subtilis* nor *Clostridium butyricum*. In addition, the *tol-1* mutants exhibited reduced leaving behavior from the *B. infantis* lawn (96).

Similarly, *daf-2* [long-lived strain (97)] or *daf-16 C. elegans* [short-lived strain (98)] mutants have been shown to have an extended lifespan similar to wild-type *C. elegans* when fed with *L. gasseri* LG2055. The mechanism allowing the increased lifespan seems independent of DAF-2 or DAF-16 since their expression as well as the expression of related genes *age-1, skn-1, sek-1*, and *pmk-1* remained stable in these conditions. However, the phosphorylation level and consequently the cellular localization (cytoplasm or nucleus) of the transcription factor DAF-16, nor the expression of the DAF-16 regulated genes have been characterized (86).

The work of Fasseas et al. showed that some strains of LAB could have a negative impact on the lifespan of the worm (Figure 1B). Adult *C. elegans* N2 worms fed with live bacterial cells belonging to the species *Lactobacillus salivarius* and *Lactobacillus reuteri* showed a significant reduction in their mean lifespan compared to those fed with *E. coli* OP50 with an average of 17, 15, and 19 days respectively. However, alive *L. salivarius* has the ability to restore a normal lifespan to short-lived *C. elegans daf-16* mutant (92) suggesting an impact of this specie to counterbalance the lack of DAF-16 function in longevity. Sugawara et al. showed that worms fed with killed *B. longum* in addition to *E. coli* OP50 displayed reduced worm's longevity in a *B. longum* dose-dependent manner. Moreover, aging worms fed with only killed *B. longum* displayed longer life span than those fed *E. coli* alone. This study revealed that killed *B. longum effects* in *C. elegans* were mediated by DAF-16 nuclear localization leading to the overexpression of DAF-16 target genes (99). These findings are in contradiction with previous observations with different *Bifidobacterium* and *Lactobacillus* strains, which showed the role for SKN-1 independently of DAF-16 (99).

Although these LAB strains have a negative impact on the wild worm's lifespan, an antitumor role in *C. elegans gld-1*^−/−^ mutant has been demonstrated. The lack of *gld-1*, coding for a tumor suppressor, causes the anarchic multiplication of cells, filling the body of the worm until its death. When adult *gld-1*^−/−^ worms are fed with *L. salivarius* or *L. reuteri*, a significant inhibition of tumor cells growth is observed with both alive and UV-killed bacteria (92, 100). Until now, the antitumor mechanism of LAB has not been elucidated even if their role in modulating the intestinal microbiota would protect against the development of colorectal cancer (101, 102). These data suggest that *C. elegans* could be used as a “living laboratory” for the study of antitumor properties of LAB or other probiotics.

Although there are both probiotic species with positive effects (11, 86, 93) and others with negative effects (92) on *C. elegans* lifespan, there are also some that have no significant effect on the nematode lifespan. These species include, for instance, *Lactobacillus helveticus* JCM1120^T^ (86) or UV-killed *Pediococcus acidilactici* (92). Considering broader probiotic strains, Kato et al. showed that both living and UV-killed *C. butyricum* MIYAIRI 588 extends the lifespan of *C. elegans* through regulation of the insulin/IGF-1 signaling (IIS) pathway and the Nrf2 transcription factor. It improves resistance to several stresses in *C. elegans* such as infections with pathogenic bacteria (*S. enterica* and *S. aureus*), UV irradiation, and the metal stressor Cu^2+^ (87). Two other bacteria from human intestine and displaying probiotic capacities, *Butyrococcus pullicaecorum* and *Megasphaera elsdenii*, extend the lifespan of *C. elegans* via the transforming growth factor-beta (TGF-β) pathway associated with anti-inflammatory processes in the innate immune system (103).

It has been demonstrated by Garsin et al. that the culture medium used for bacterial growth had a significant importance on the viability tests. *E. coli* cultured on brain heart infusion broth (BHI) leads a fast mortality to the nematode with a lethality time 50% (LT50) of only 6 days whereas it is not particularly pathogenic on Nematode Growth Medium (NGM). The authors also stated that two other species, *Enterococcus faecium* and *Streptococcus pyogenes*, do not show any significant worm mortality on BHI whereas this may be the case using NGM (33). Even if it is the only published work on this subject, this critical experimental point must be considered when working on the probiotic effect of microorganisms.

Although represented in an ultra-majority by lactobacilli and bifidobacteria, probiotics should not be limited to only prokaryotes. Indeed yeasts can also play this role. The recent study by Veisseire and colleagues has thus demonstrated, in *C. elegans*, the probiotic properties of the cheese yeast *Debaryomyces hansenii* Dh25 (104). The authors showed that in its presence, the nematode had a significantly increased life expectancy and was able to resist to a *S*. Typhimurium UPsm1 infection. Additional studies are nevertheless necessary to characterize the mechanisms involved. However, such studies are promising since it strengthens the antipathogenic arsenal represented by probiotics.

Nowadays, although the health benefits on higher animals by probiotics are indisputable, there is no proof of an improvement of the life expectancy induced by these microorganisms except with the nematode (84). Considering that the pathways that influence aging and longevity are well-conserved among eukaryotic species from yeast to mammals, including *C. elegans* (105, 106), the use of this model to evaluate the potential benefits of microorganisms on human health during aging and longevity and to understand the host-microorganisms interactions is promising. The Insulin/IGF-1 signaling (IIS) pathway is in the focus of discussions concerning *C. elegans* longevity and health span. Further investigations are needed to determine if long-lived worms with reduced IIS (*daf-2* mutants) enjoy a better health related to their extended lifespan and to understand how is aging delayed in *C. elegans*. Even if the *C. elegans*-*E. coli* OP50 binary model does not show the same relationship than human share with its microbiota complex community, it allows the study of bacterial species for their direct or indirect role in the nematode physiology and longevity. *C. elegans* offers the possibility of carrying out screening tests on collections of microorganisms to envisage the selection of new strains with probiotic potential. These new knowledge would allow the setting up of mechanistic hypothesis for further investigating in mammalian models or on human and of new paradigms applicable to more complex organisms such as humans (107).

### Mechanisms and Pathways Implied for Worm Longevity

In *C. elegans*, the regulation of life expectancy mainly involves at least three signaling pathways: DAF-2/DAF-16 (insulin-like signaling pathway), p38 MAPK, and JNK pathways. In the DAF-2/DAF-16 pathway, activation of the DAF-2 transmembrane receptor induces a phosphorylation cascade via AGE-1/PI3K (phosphoinositide 3-kinase) and the Serine-Threonine kinases (PDK-1, AKT-1, AKT-2) which regulates the DAF-16/FOXO transcription factor by phosphorylating it. When DAF-16 is dephosphorylated, it is translocated into the nucleus where it activates the transcription of genes involved in lifespan control (108).

The p38 MAPK (also called PMK-1) pathway is activated through phosphorylation by upstream MAPKK (SEK-1), which is activated by MAPKKK (NSY-1). It has a role in regulating life expectancy by modulating the specific immune response of the worm and has been shown to be required in its resistance to bacterial infection (109). Under the control of PMK-1, potential antimicrobials are secreted such as C-type lectins, ShK toxins, or CUB-like genes in response to *P. aeruginosa* infection. In addition, a complete genome expression analysis suggests that the DAF-2/DAF-16 and p38 MAPK pathways are two distinct pathways since they do not positively regulate the same genes at the same time (29). Another study showed that PMK-1 contributes to the increased longevity of *daf-2* mutants and that this increased longevity was mediated by DAF-16 via the upregulation of antimicrobials (110). PMK-1 may play a similar role via distinct immune effectors to enhance lifespan suggesting that innate immunity is a key determinant of longevity. Because immunity is tightly associated with human longevity as well, further characterizing the interplay between immunity and longevity in *C. elegans* may provide us new insights into the mechanisms of human aging and age-related diseases.

The JNK (c-Jun N-terminal Kinase) pathway is activated by cytokines and in response to stresses such as UV irradiation, Reactive Oxygen Species (ROS), DNA damage, heat stress, and inflammation (111, 112). In *C. elegans*, this pathway evolves in parallel with the insulin signaling pathway till it converges to DAF-16 which is phosphorylated by JNK-1 (28, 113). The activation of JNK-1/DAF-16 signaling pathway leading to the overexpression of *sod-3* (superoxide dismutase) gene has been shown to be activated by *B. longum* BB68 to increase the longevity of the nematode (94).

In the case of *B. subtilis* NCIB3610, several mechanisms explaining its beneficial effects on *C. elegans* have been proposed. It has been found that one of the key conditions to promote longevity of the worm was the formation of a biofilm in the intestinal environment. The expression of *bslA* gene and the *tapA-sipW-tasA* and *epsA-G* operons, encoding essential components to the biofilm formation (Hydrophobin, TasA protein, and the EPS exopolysaccharide, respectively), were identified as necessary for extending the worm's longevity (114). The second mechanism is based on the use of bacterial nitric oxide (NO) to mediate lifespan extension via the expression of a group of genes that function under the dual control of HSF-1 (an activator of heat shock proteins) and DAF-16 transcription factors (115). *C. elegans*, which is unable to synthesize NO because it lacks NO synthase, uses the one produced by the bacteria to regulate its own metabolism (114, 116). NO provides better thermotolerance due to activation of HSF-1 (115). Another study focused on *Lactobacillus fermentum* MBC2 highlighted the role of fat metabolism in *C. elegans* viability, further supporting the strong correlation between reduction of fat storage and extension of lifespan in nematodes. They showed a PEPT-1-dependent pro-longevity effect, mediated by the transcriptional factor DAF-16; *pept-1* gene is one of the major regulators of fat content in *C. elegans* (76). The measure of the ratio of lipids and proteins in the worms could be informative to evaluate the potential beneficial effect of a LAB strain on its lifespan.

Recent studies suggested a key role of bacterial quorum sensing molecules in the repression of *C. elegans* infection with the pathogens *Candida albicans* or *P. aeruginosa*, showing that the nematode can detect microbial signals and develop a symbiotic interaction (117, 118). The quorum-sensing pentapeptide CSF (Competence Sporulation Stimulating Factor) of *B. subtilis* has been shown to play a role in the extension of the nematode longevity (114). *B. subtilis* CSF was reported to contribute to intestinal homeostasis of the host by activating the p38 MAPK survival pathways and by inducing the synthesis of cytoprotective heat shock proteins (HSPs) (119, 120). The nematode survival may be due to quorum sensing molecules such as NO and CSF and to biofilm formation (114).

All this data highlighted the idea that LAB strains can exert a beneficial effect on *C. elegans* via several specific mechanisms and pathways. *C. elegans* can be considered as a pertinent model for the screening of potential lifespan enhancing probiotics and their anti-aging compounds.

## Protective Effects of LAB Against Abiotic Stress in *C. elegans*

The environment in which *C. elegans* evolves can be the source of abiotic stress (oxidative, thermal) having a harmful effect on the physiology of the animal. The scientific community has therefore wondered if the administration of LAB could allow the nematode to survive to these stresses with the aim to further understand the mechanisms involved in abiotic stress-resistance.

### Reactive Oxygen Species and Hormesis

ROS are responsible for oxidative damage to proteins, lipids as well as to nucleic acids leading to severe mutations in DNA (121, 122). A close association exists between ROS generation, ROS-related damage and aging (123). Reducing the amount of Reactive Oxygen Species (ROS) is essential to maintain a good health in most living organisms. In some model organisms such as the yeast *Saccharomyces cerevisiae*, the fly *D. melanogaster* or the worm *C. elegans*, low doses of ROS seem to induce promotion of health and lifespan whereas higher doses are deleterious (76, 124).

**Figure 2:**
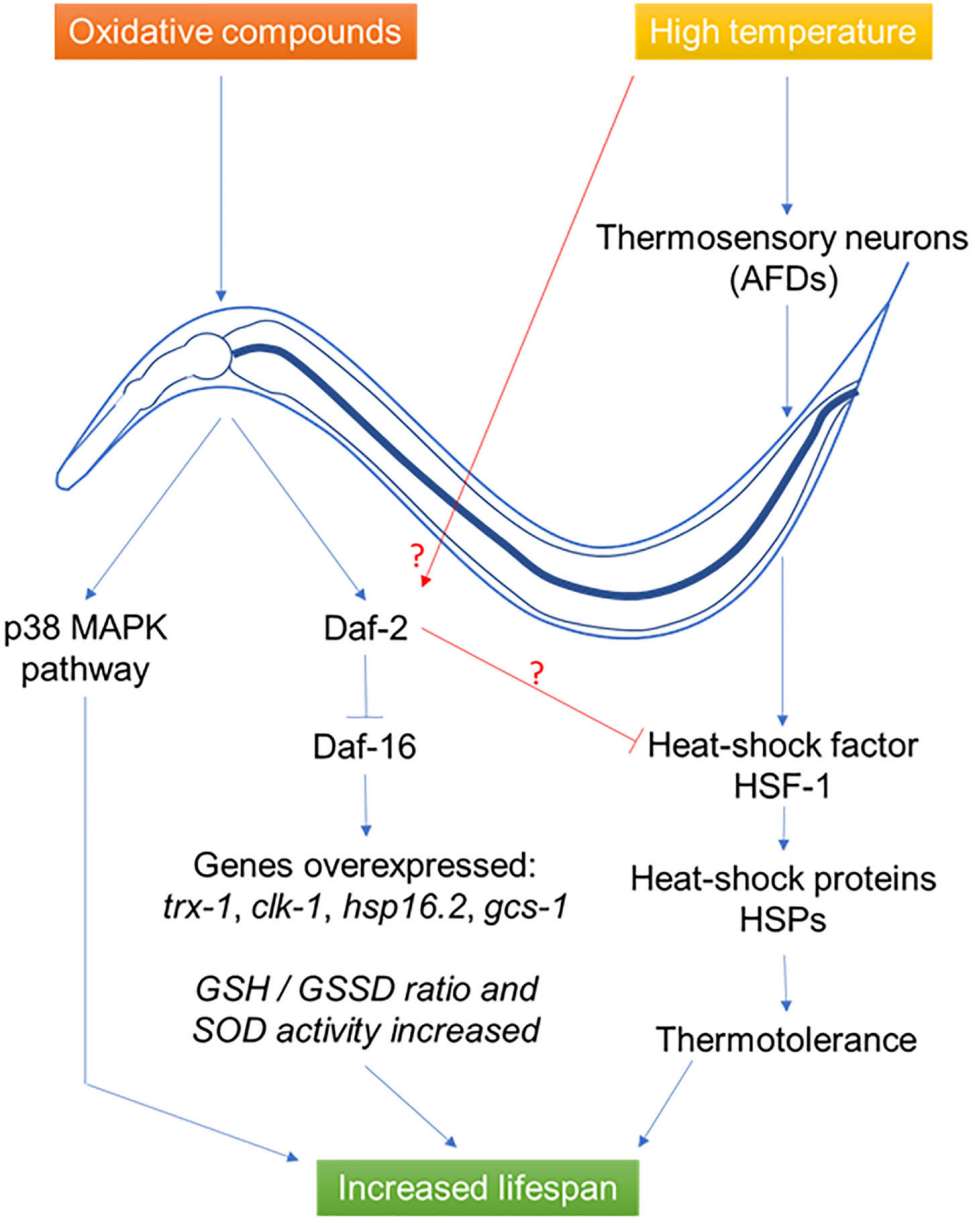
Effects of probiotics *L. gasseri* SBT2055 (86), *L. rhamnosus* CNCM I-3690 (11), *B. longum* (99), and *B. subtilis* NCIB3610 (114) on *C. elegans* during abiotic stress. During oxidative stress, the bacteria induce a response in the host via the p38 MAPK and DAF-2/DAF-16 pathways allowing the synthesis of antioxidant metabolites. In the case of thermal stress, a neural response induces the synthesis of heat-shock proteins to allow thermotolerance. In both cases, the result is an increase in the longevity of the host.

This phenomenon i.e., the biphasic response to a potentially harmful compound, firstly described in 1943 by Southam and Ehrlich (125) under the name of hormesis, then to mithormesis (i.e., mitochondrial hormesis), has been discovered more recently in *C. elegans* (126). It was shown that many of the Insulin/insulin-like growth factor signaling pathway genes had a role in hormetic processes, either in response to heat stress (e.g., *daf-3* and *daf-5*), or in lifespan extension (e.g., *daf-12* and *daf-16*) or both (*daf-18)* (Figure 2). Regarding the worm, the study was focused on gene expression and thus the physiological and metabolic effects arising from thermal stress remain uncharacterized. A study showed that ROS signals are transduced mainly by the oxidative stress transcriptional factors SKN-1 (Nrf2 or NFE2L2 in mammals) and JUN-1, and partially by DAF-16. Cell biology experiments demonstrated a similarity between human DUOXs and *C. elegans* BLI-3 dual oxidases, suggesting that DUOXs are potential targets of intervention for lifespan extension. They suggest that low levels of ROS, fine-tuned by the peroxidase and dual oxidase, act as second messengers to extend lifespan by the effect of hormesis (127).

### Oxidative Stress

One of the indicators of the presence of a possible oxidative stress is the reduced glutathione form (GSH)/oxidized form (GSSG) ratio. Under normal physiological conditions, the reduced form predominates, in order to be subsequently oxidized by ROS if necessary (128). *L. gasseri* SBT2055 is at the origin of a 3-fold increase of the GSH/GSSG ratio compared to *E. coli* OP50 condition. Combining the increase in SOD activity and a greater amount of reduced glutathione, *L. gasseri* SBT2055 has been shown to play an important role in reducing ROS and damage related to oxidative phenomena (86).

*L. gasseri* SBT2055, a lactic acid bacterium previously mentioned for its ability to increase the lifespan of the nematode, also allows the host organism to fight effectively against oxidative stress. Analyses of 10-day-old adult worms fed with *L. gasseri* SBT2055 showed a significant overexpression of *sod-*1 gene and an 80% increase in superoxide dismutase activity compared to worms fed with *E. coli* OP50 as well as an enhanced activity of the Nrf-2 ortholog SKN-1. The same molecular anti-oxidative mechanism has been identified in mammalian cells, protecting cells against oxidative stress (129). Many other genes have been reported to have significant over-expressions such as *trx-1, clk-1, hsp16.2*, or *gcs-1* respectively encoding a thioredoxin, a mitochondrial polypeptide, a heat-shock protein and an ortholog of the γ-glutamyl-cysteine synthetase (86).

*L. rhamnosus* CNCM I-3690, in addition to its beneficial effects of the worm's lifespan, gives *C. elegans* a protection against oxidative stress. This strain causes activation of DAF-16 transcription factor, which subsequently induces an increased longevity, a decrease in the amount of lipid inclusions and an increased resistance to hydrogen peroxide. These three effects are the result of the reduction of the inflammatory process. In addition, similar results were observed *in vitro* using dendritic cells culture where *L. rhamnosus* was actively involved in reducing pro-inflammatory cytokine ratios such as IL12/IL10, IL6/IL10, IL8/IL10, and TNFα/IL10 (11). Another study showed that worms fed with killed *B. longum* in addition to *E. coli* OP50 had a higher survival rate, compared with worms fed *E. coli* alone, following hydrogen peroxide-induced oxidative stress and heat stress at 35°C (99).

### Heat Stress

*C. elegans*, like most living species, is sensitive to temperature and can set up various defense strategies. A whole range of highly conserved genes, under the control of the two thermosensory neurons (AFDs), are activated (130) to restore cellular homeostasis when the organism detects a rapid rise in temperature (131). The lactic acid bacteria *L. gasseri* SBT2055 allows significant resistance of *C. elegans* to heat stress, without the underlying mechanism being evoked (86).

*B. subtilis*, although not part of the lactic acid bacteria group, possesses interesting probiotic capabilities. A recent study concerning *C. elegans* resistance against heat stress showed that the strain *B. subtilis* NCIB3610 induced a lifespan 132% higher on average compared to worms that were fed with standard *E. coli* OP50 strain. Similar results have also been observed in the case of osmotic and heavy metal stresses (114).

Concerning these abiotic stresses, some authors hypothesized that the probiotic may have a similar effect in mammals but, even if signaling pathways are well-conserved, the possibility to extrapolate this data from nematodes to mammals has to be investigated (11, 129, 132).

## Influence of LAB on *C. elegans* Immunity

**Figure 3:**
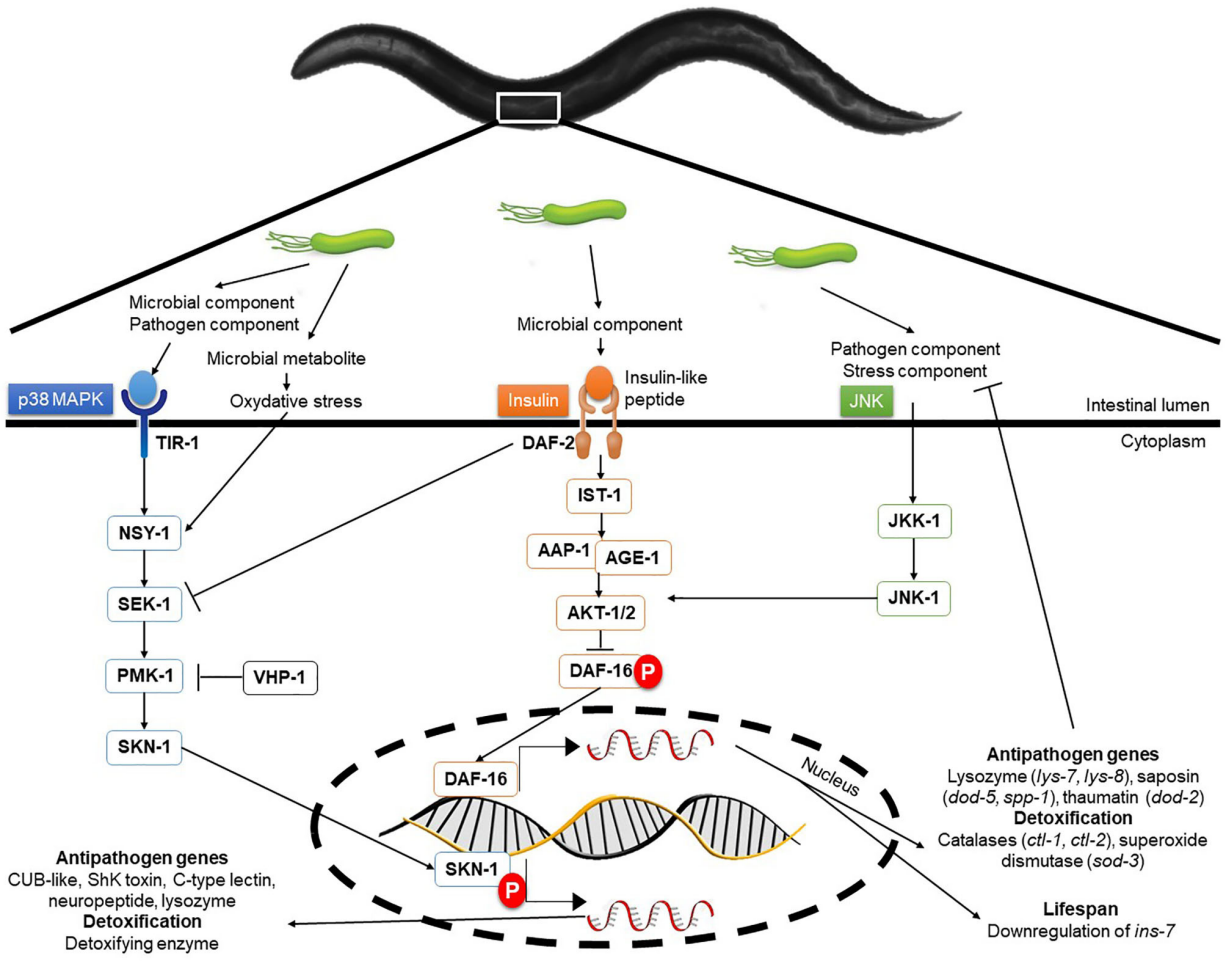
Molecular interactions and signaling pathways involved in *C. elegans* immune response. In the nematode, three highly conserved signaling pathways are the basis of the immune response protecting the animal from biological and abiotic aggression. These pathways are the p38 MAPK, the DAF-2/Insulin-*like* receptor and the JNK pathways. Interconnections and back-controls exist in these signal cascades and allow for fine control of the defense mechanisms put in place in the face of aggression. Transcriptomic analyses have revealed the absence of overlapping in the expression of genes regulated by these pathways.

The protection of an organism against a pathogen, whether it be a mammal, a bird or a nematode, involves highly conserved signaling pathways (110). In *C. elegans*, two major signaling pathways (Figure 3) are involved in the innate immune response: the mitogen-activated protein kinase (MAPK) pathways and the Insulin/IGF1 Signaling (ILS) pathway or DAF-2/Insulin-like Receptor (DIR) (133).

MAPK pathways, considered to be the oldest transduction pathways of immunity (134), combine several types of cascades in *C. elegans*. The main, p38 MAPK, originally described for its action against Gram-negative bacteria, is also described for Gram-positive and fungi (133, 135). Two genes, essential for resistance to pathogens, have been identified namely *sek-1* and *nys-1*, respectively coding for a MAP kinase kinase (MAPKK) and a MAP kinase kinase kinase (MAPKKK) (134). The probiotic *L. fermentum* JDFM216 is able to induce the over-expression of genes from C-lectin family, including *clec-60* and *clec-78*, as well as F-box protein and NHR related genes, as a consequence of PMK-1 signaling activation (34). Therefore, it seems that this bacterium can induce an immune response in the nematode during a possible infection. It has been shown that p38 MAPK is involved against cytolytic pore-forming toxins such as *Bacillus thuringiensis* Cry5B toxin (136).

The second main pathway, the DAF-2/Insulin-like receptor (DIR) pathway involving the transcription factor DAF-16, also plays a major role in the immune response although this is not fully understood (133). Transcriptomic analysis revealed DAF-16 promoted the expression of genes linked mainly to longevity and to defense against pathogens (137, 138).

Parallel to the DAF-2/DAF-16 pathway, the c-Jun N-terminal kinase (JNK) pathway is activated by environmental stresses and inflammatory cytokines and acts as a molecular sensor for the pathway DAF-2/DAF-16 (139). Some constituents of the cell wall of lactic acid bacteria, such as muramyl dipeptide (molecule on the basis of the parietal peptidoglycan), are involved in the activation of the immune response and in particular the synthesis of cytokines (TNFα and IL-1 in mammals) by immunocytes (84). These cytokines are activators of c-Jun N-terminal kinase (JNK) pathway, which is a DAF-16 positive regulator (113).

*C. elegans* presents different transcriptional responses depending on the fungal or bacterial infection origin (116, 140). The genes *fil-1* and *clec-71*, respectively encoding for a lipase and a C-type lectin are upregulated by both *P. aeruginosa* and *S. aureus* but not by *C. albicans*. Gram-positive and Gram-negative bacteria also induce a separate response i.e., in the presence of *P. aeruginosa*, there will be an overexpression of *crp-3* (peptidase), *cyp-35A2*, and *cyp-35C1* (cytochrome P450) genes while in the presence of *S. aureus, pmp-1* (ABC transport), *gst-28* (glutathione S-transferase), and *lys-5* (lysozyme) genes are overexpressed. There is a common immune response to all three genera *Candida, Staphylococcus*, and *Pseudomonas* including the induction of the *far-7* (nematode fatty acid retinoid binding) and *cyp-37B1* (cytochrome P450) genes whereas *sams-1* (S-adenosylmethionine synthetase), *acdh-1* (acyl-CoA dehydrogenase), and *nhr-114* (ligand binding domain of nuclear hormone receptor) genes are repressed (116, 140). Another study showed that *nhr-86* induces innate immune defense independent of the p38 MAPK *pmk-1* in response to *P. aeruginosa* (141). Finally, a study demonstrated that *C. elegans* response to infection by *B. thuringiensis* showed significant profile changes at the level of proteome, such effect being under the influence of AMP-activated protein kinases (AMPKs) which were supposed to be regulators of the worm immune response (82). *C. elegans* has neither phagocytic cells nor an adaptive immunity. Comparing to human, the single toll-like receptor does not seem to play a role in the immune response and the nematode has no NF-κB-like factor (34, 142). Anyway, because of the great conservation of the signaling pathways involved in innate immunity across species, *C. elegans* could be a tool of interest for the molecular characterization of immune pathways. Complementary approaches such as the use of *C. elegans* mutants and transcriptomic will allow the analysis of the immune pathways that are modulated by LAB.

## LAB Anti-pathogen Effects

In nature, *C. elegans* is found in numerous ecological niches and is likely to be host to yeasts like *Candida* or to bacteria such as *Pseudomonas, Enterococcus* (116), *Micrococcus luteus, Bacillus megaterium* (143), or *Comamonas aquatica* (144). The greater majority of worms have their intestine colonized by bacteria, especially those fed *in vitro* with *E. coli* OP50, as early as 2 days of adulthood (145).

*S. enterica* serovar Enteritidis, a pathogenic enterobacterium for humans, is capable of inducing mortality in *C. elegans* in just a few days (40% in 5 days) after invading its intestine. However, the young adults (3 days) are resistant both to the colonization of their intestine by *Salmonella* and to its pathogenicity compared to the 5-day-old worms. A resistance to infection was highlighted in elderly adults (7 days) fed with probiotic bacteria (*L. helveticus, L. plantarum, L. rhamnosus, B. infantis*, and *B. longum*) before infection by the pathogen. It is interesting to note that the pathogenicity of *S*. Enteritidis in the nematode requires alive bacteria; otherwise, *C. elegans* has a lifespan quite like that encountered with *E. coli* OP50. *L. rhamnosus, plantarum*, and *helveticus* have been reported providing a much better resistance to *S*. Enteritidis infection in the worm without limiting its presence in the intestine. Consequently, the mechanism of inhibition of the virulence of *S*. Enteritidis would not be due to the synthesis of antimicrobial compounds but rather to a certain immunotolerance (84). Rangan et al. showed that the antigen A SagA secreted from *E. faecium* is sufficient to promote pathogen tolerance in a *tol-1* dependent manner, protecting *C. elegans* against *Salmonella* pathogenesis (132). This protective activity has been observed in mice as well. In worms fed *E. faecium* L11, the increased expression of genes related to the MAPK pathway (*pmk-1* and *sek-1*) and to the TGF-β pathway (*dbl-1* and *sma-3)* was shown to contribute to the resistance against *Salmonella* infection (35). Lysozymes, such as LYS-8 which is under the control of *dbl-1* and *sma-3*, and lectins were identified as antimicrobial peptides that help to nematode defense against infection (146). Recently, a study has focused on the antimicrobial properties of the probiotic strain *E. coli* Nissle 1917 (EcN) against infection by enteropathogenic *E. coli* (EPEC). The authors showed that EcN inhibited the pathogenicity of EPEC by competitive colonization of *C. elegans* intestine. In addition, they also demonstrated that EcN enhances the integrity of the intestinal barrier via the induction of Zonula occludens ortholog (Zoo-1), a tight junction protein (147). A transcriptomic study showed that *Pediococcus acidilactici* P25 affects the expression of genes relative to innate immune response, peroxisome, longevity, and MAPK pathways and reduced the gut colonization of the worm and the expression of enterotoxin genes by *E. coli* ETEC K88 (148). In addition, the work of Chelliah and colleagues has shown the benefit of using antimicrobial peptides isolated from the cell-free supernatant of *Pediococcus* to treat bacterial infections. Indeed, after preincubation with a molecule produced by *P. acidilactici* and molecular weight lower than 3 kDa (pediocin), nematodes showed increased survivals facing *E. coli* O157 and *Helicobacter pylori* infections. This increased survival was also associated with a significant decrease in the bacterial load in the gut of the worms (149).

The pathogen *C. albicans* ATCC® 10231™ can induce a 100% mortality in *C. elegans* in 3 days. *L. rhamnosus* Lcr35®, in addition to having a pro-longevity activity, protects the worm from a *C. albicans* infection (+267% of survival) even if the yeast is still detectable in its intestine. At the mechanistic level and during a preventive treatment, genes of the p38 MAPK signaling pathway are repressed and genes involved in the antifungal response are induced by Lcr35®, suggesting that the pathogen appears not to be detected by the worm immune system. However, the DAF-16/FOXO transcription factor, implicated in the longevity and antipathogenic response of *C. elegans*, is activated by Lcr35®, suggesting that the probiotic strain acts by stimulating its host *via* DAF-16 but also by suppressing the virulence of the pathogen (90). However, in a second study, the authors showed for the first time the effectiveness of a Live Biotherapeutic Microorganism (i.e., *L. rhamnosus* Lcr35®) in a curative context against candidiasis and a different mechanism of actions with, the activation of the p38 MAPK pathway in the nematode (150). It has been also demonstrated that molecules involved in bacterial quorum-sensing such as indole and its derivative, indole-3-acetonitrile (IAN) were able to significantly alleviate the virulence of *C. albicans* in *C. elegans* reducing the fungal burden in the intestine (118). In another study, a clinical isolate of *L. paracasei* increased the survival of *C. elegans* infected with *C. albicans* by 29%. This prolonged longevity was accompanied by the inhibition of filamentation of *C. albicans* ATCC® 18804™, preventing the cuticle rupture of the worms of 27%. A negative regulation of the *tec1* and *ume*6 genes that are essential for the production of hyphae was suggested to explain the reduced filamentation of *C. albicans* (151). However, the authors have not demonstrated the effect of *L. paracasei* on the immune response of the nematode.

With regard to colonization of the intestine, the prerequisite that one condition for a microbial strain to be recognized as probiotic is its ability to colonize the gut (if used orally) is controversial (152, 153). In spite of the fact that *L. gasseri* SBT2055 had undeniable positive effects on the worm and that its presence in its intestine had been confirmed, it is unable to colonize it (86). It would be of interest to follow *L. gasseri* STB2055 population to specify whether it constitutes a transient microbiota or if it is destroyed in the digestive tract. It has also been shown that *L. plantarum*, as well as *L. zeae* LB1 and *L. casei* CL11, was able to colonize the *C. elegans* intestine and to remain on it over 4.3 CFU/mL per worm (this is higher than 3.2 CFU/mL per worm obtained with *L. rhamnosus* GG), thereby contributing to the inhibition of *S. aureus* by stimulating the innate immune system but the underlying mechanisms are not clearly defined (74). Another study evaluating 15 probiotic strains including *L. plantarum* K90 and *L. paracasei* CD4 that exhibited increased nematode lifespan showed that gut colonization ability differs among the strains tested (154). The last study of Sharma suggests that the adhered probiotic strains inhibit the attachment of *E. coli* pathogenic strains on the intestinal layer of *C. elegans*, blocking receptors sites and reducing pathogen colonization (89). A study on 35 strains of LAB showed the ability of three strains, *L. brevis, P. acidilatici*, and *P. pentosaceus*, to increase the lifespan of *C. elegans* infected with *P. aeruginosa* PA14 and to colonize the *C. elegans* gut (32). In the Ikeda et al. study, lactic acid bacteria have not been able to reduce the colonization of the intestine by the pathogen which may be the sign of the absence of synthesis of antimicrobial compounds (84). This lack of inhibition of colonization of the gut by a pathogen has also been demonstrated with enterotoxinogenic *E. coli* (ETEC), a deadly strain for *C. elegans*, in the presence of *L. zeae* LB1 and *L. casei* CL11. In the present case, the protection of the worm by the probiotics is due to the inhibition of the enterotoxins synthesis whose mechanism is not yet understood (155). Both lactobacilli, as well as the *L. plantarum* JDFM LP11 and the *L. rhamnosus* GG (36) are able to colonize the intestine of the worm and are found at the rate of 10^4^ to 10^5^ CFU/worm, as much as for ETEC. The relevance of intestinal colonization for a probiotic strain to exert its optimal effect must be further characterized in the worm before to be studied in mammals and extrapolated to human.

The enterohaemorrhagic *E. coli* (EHEC) O157:H7 strain also exhibits significant pathogenicity to *C. elegans*, thereby reducing its lifespan compared to an avirulent bacterium. A study using the probiotic *E. faecalis* [marketed under the name Symbioflor® (Symbiopharm, Herborn, Germany)] showed that *E. faecalis* exerted a protective effect, significantly reducing the mortality of *C. elegans* due to EHEC. A mix *E. faecalis*—*E. coli* O157:H7 restored partly a lifespan but not as that observed with *E. coli* OP50 or Symbioflor® alone. It is of interest to note here the potentialization of the *E. faecalis* strain anti-EHEC effect by the galenic Symbioflor®. This beneficial effect of *E. faecalis* was explained by the fact that the probiotic negatively regulates a large number of genes associated with the virulence of *E. coli* O157: H7 like *espA, espB, espD* (encoding for LEE structural element), or *sepQ* (pore forming protein) (156). However, the *stx1B* and *stx2B* genes both encoding for Shiga-like toxin subunits are not repressed in the presence of the probiotic (156), as was the case with *L acidophilus* (157). The authors made the hypothesis that the inactivation of the toxin can be explained by the fact that both *E. coli* and *E. faecalis* are lysed by the grinder of the worm and that the fragments of *E. faecalis* inhibit the fixation of toxins on cellular receptors (156). At the early infection by enterohaemorrhagic *E. coli*, the p38 MAPK pathway, involved in the innate immune response is induced by Stx-1 toxin (158). In the case of *L. pneumophila*, it appears that when adult *C. elegans* is fed with *B. infantis* and then exposed to *L. pneumophila*, a significantly increased resistance to the pathogen was observed although *B. infantis* did not reduce the amount of the pathogen in the gut. This strengthening of the immune system would imply, as for longevity, the PMK-1/p38 MAPK signaling system (159).

In addition, opposite bacterial responses were highlighted. Feeding *C. elegans* with *L. acidophilus* will result in protecting the worm against infections of pathogenic Gram-positive *E. faecalis* and *S. aureus* but not against *P. aeruginosa* (160) which is a Gram-negative bacterium. Moreover, exposing *C. elegans* with a non-pathogenic Gram-negative bacterium, *P. mendocina*, will have a positive action against infections of the pathogen *P. aeruginosa* PA14 (116). All these data show that the beneficial or deleterious effect of a bacteria on *C. elegans* remained strain dependent leading to suggest that a characterization of the interaction mechanisms are needed. A question is then arising concerning these contradictory effects of the same strain for future applications on human health.

In conclusion concerning the anti-pathogen effects of probiotics observed in *C. elegans*, it looks like only part of the signaling pathways are characterized. The molecular mechanisms underlying the interactions between probiotics, pathogens and hosts have yet to be explored. Moreover, the role of probiotics gut colonization in the resistance to pathogens is not clearly evaluated and would be strain dependent. A strain-by-strain molecular study using complementary approaches on *C. elegans* appeared to be necessary to highlight the probiotic potential of a microorganism. Another application using the worm would be to test the anti-pathogen potential of microbial consortia (bacteria and fungi) rather than single microorganism. The data obtained would help us to understand the link between microbiota and health i.e., anti-pathogen effect, aging, immunity and to design strategies to fight against a large panel of pathogens. Moreover, *C. elegans* seems to be less susceptible than mammalian cells to antibiotic drugs targeting the lipid-bilayer membrane of the pathogenic microorganism *S. aureus* (34, 142). This point concerning the host cell physiology has to be considered when evaluating the anti-pathogen potential of a new probiotic strain dedicated to human health. It also seems important to be interested in the impact of probiotics on the physiology (general morphology, structural integrity) of the nematode after the ingestion of these microorganisms, in a qualitative (microscopy) and quantitative way by methods of transcriptomics or proteomics (i.e., expression and translation of cuticle proteins). Finally, it is necessary, through mechanistic studies, to precisely establish the link between the immune response, the effective defense against a pathogen and other fundamental biological functions such as longevity.

## Translational Research and Prospects

Understanding the mechanisms of action of a microorganism in a host such as *C. elegans* has led to many technological and scientific breakthroughs. However, the opportunities of transposition to the human model are mandatory in order to develop therapeutic strategies dedicated to human health.

### The Roundworm as a Model System to Study Human Diseases

Molecular decoding of host-microorganisms interactions mechanisms requires the implementation of *in vitro* and *in vivo* approaches to investigate their biochemical, genetic, and phenotypic components. A better understanding of genes and their interacting partners forms the foundation for translational research leading to mechanism-based therapeutic strategies (161). In absolute terms, studying humans to understand the molecular mechanisms of human disease could be considered as the ideal solution. However, Humans are poor subjects for these studies because of heterogeneous genetic backgrounds, low reproductive rate, generation times far too important for experimental studies, wide variations in gene expression, subject to strong environmental variations and experimentations on humans are unethical. To circumvent these issues, *C. elegans* is being exploited to decipher human biology thanks to its properties such as the availability of molecular tools and genome sequenced and annotated, the availability of a bank of mutants, crossbreeding facilities to study genetic, high reproducibility rate and homologies with humans (161). The number of human disease-related genes that share homology with *C. elegans* genes ranges from 40 to 75% (162). Even in the absence of an orthologous gene, it is likely that there are protein domains or parts of biochemical pathways in *C. elegans* that are sufficiently conserved to allow the extrapolation of the mechanisms from the nematode to a model of human pathobiology (163).

Although *C. elegans* is a bacterivorous organism, these animals are killed by a variety of human pathogens including Gram-negative (*P. aeruginosa, S. enterica, Serratia marcescens, Burkholderia cepacia*) and Gram-positive organisms (*S. aureus, Streptococcus pneumoniae, E. faecalis*) as well as fungi (*Candida* species) (33, 84, 140, 150, 160). Several studies show that many of the host resistance (e.g., antimicrobial peptides (AMPs), MAPK signaling cascades) and bacterial (and fungal) virulence factors (e.g., *Pseudomonas* exotoxin A, *Staphylococcus* V8 protease) that operate in *C. elegans* infection models are the same in plants, arthropods and mammals. Thus, *C. elegans* infection models can serve as a useful system for dissecting apart the molecular components of host-pathogen interactions. Although evolutionarily distant from humans, *C. elegans* and humans share many conserved cellular pathways. Using a forward genetics screen for genes that enhanced susceptibility to pathogens, *C. elegans* was shown to possess several well-defined MAPK signaling cassettes [including those associated with PMK-1 (p38), KGB-1 (JNK), and MPK-1 (ERK)] that are crucial for the innate immune defense (164) and are found in arthropods and mammals. Taken together, genetic mutant analysis, transcriptional profiling and biochemical studies in *C. elegans* show that MAPK signaling is one of the primordial, yet still essential, stress response pathways in eukaryotes (165). Notably, insulin and TOR pathways function in *C. elegans*, as in other organisms, to coordinate nutrient and metabolic state with cellular processes (166). A better delineation of these signaling pathways should enhance our understanding of innate host resistance to a range of human pathogens. Nutrition is paramount in shaping all aspects of animal biology. In addition, the influence of the intestinal microbiota on physiology is now widely recognized. Given that diet also shapes the intestinal microbiota, this raises the question of how the nutritional environment and microbial assemblages together influence animal physiology. Leulier et al. proposed an integrative framework within which to define the study of the diet-physiology-microbiota systems and ultimately link it to human health (167). *C. elegans* are bacterivorous that can survive on a variety of bacterial species. For *C. elegans*, bacteria are both diet and microbiota. These bacteria influence metabolism, life history traits and gene expression (143, 144).

Taken together, these studies confirmed that live whole animal screens using *C. elegans* could be used as a biologic platform for potential probiotic strain discovery and characterization.

### Challenges and Opportunities

**Figure 4:**
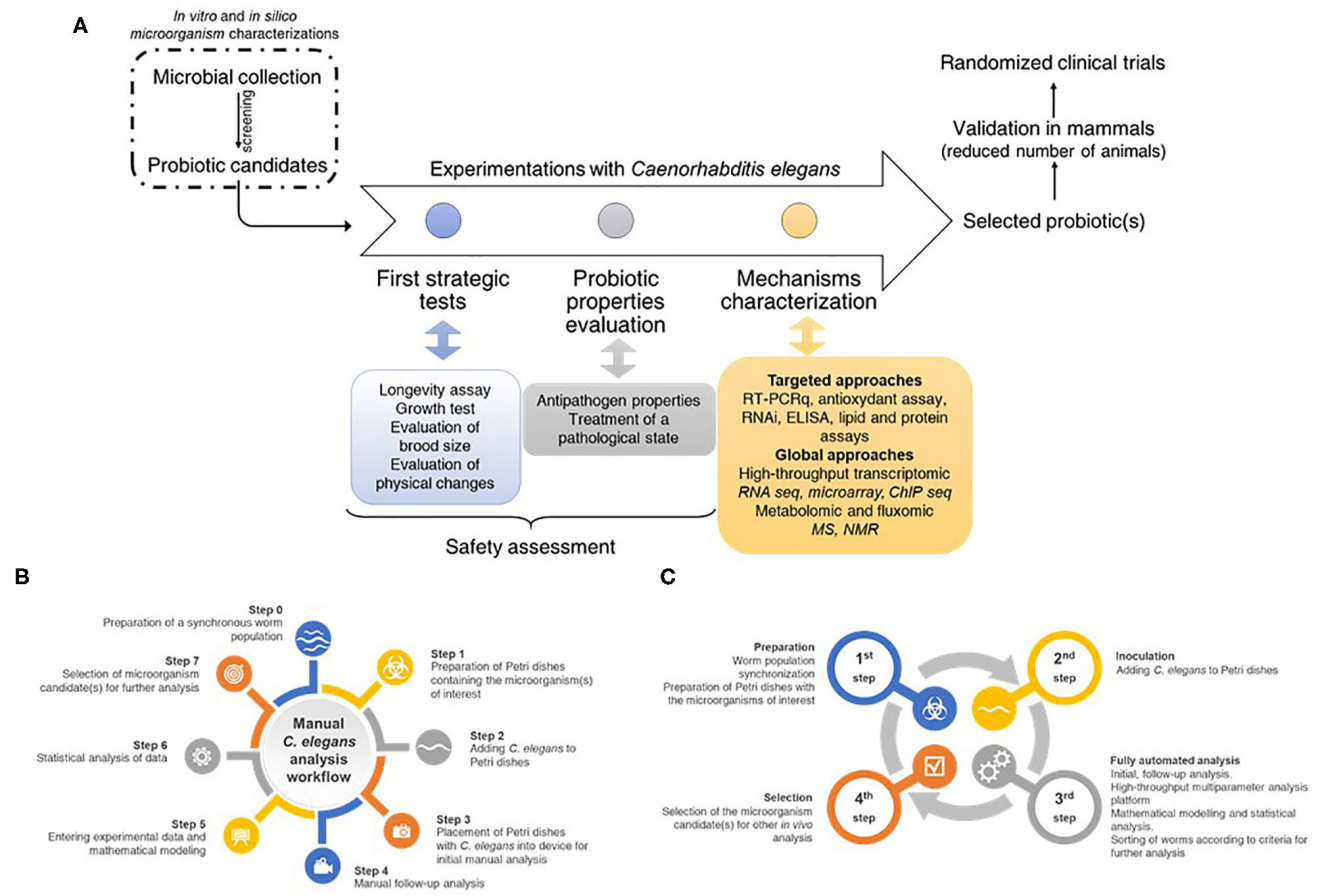
**(A)** Strategy and workflow for screening, selecting, and studying probiotic microorganisms using an integrated process. A collection of microorganisms of interest must be tested by conventional *in vitro* methods to select strains that meet the eligibility criteria defined in the literature. These candidates are then characterized using the *C. elegans* model to describe their physiological and molecular characteristics to move on to the next steps. These consist of the establishment of preclinical models (i.e., rodents) and then clinical trials in humans before a possible marketing as a drug (or any other galenic form). **(B)** Manual *C. elegans* analysis workflow representing the numerous steps required by manual experiments. **(C)** Full automated *C. elegans* analysis workflow allowing a reduction in the number of steps, a reduction in the time required for the experimenter as well as biases inherent in handling errors.

Most studies using *C. elegans* as an *in vivo* model concern the anti-pathogenic effect of bacterial strains that could be used to fight against human pathogens but the major challenge is now to go further insight the mechanisms involved in the antimicrobial capacities of beneficial microorganisms. These studies have been facilitated by the discoveries of natural microbes that infect *C. elegans*, including bacteria, fungi, and viruses. Many of these microbes share a common site of infection, the *C. elegans* intestine that is the way of exposure of many pathogens as in higher eukaryotes. *C. elegans* possesses many of the characteristics of an efficient model for the characterization of a probiotic strain and to define host-microbes and microbes-microbes interactions. It is a powerful and easy-to-use model already used to evaluate the impact of microorganisms on *C. elegans* longevity, immunity and to study their anti-pathogen effect. It offers the opportunities to investigate link between innate immunity and aging as well as between intestinal microbiota and aging. Moreover, the *C. elegans* model can be used to evaluate the safety of microorganisms, evaluating its lifespan (168). The challenge to validate *C. elegans* as a relevant model to study strains safety is to evaluate how can be made the parallel with humans (169). *C. elegans* offers good opportunities to make this link because of its strong similarity between worm and human intestinal cells in terms of physiology and functionalities. This model can be used to study a large panel of pathogenic and probiotic microorganisms with the aim to further investigate human disease mechanisms. Moreover, novel approaches are needed to develop microbial cocktails targeted to restore dysbiotic states in microbiota-associated human diseases (170). In the coming years, *C. elegans* could be added in screening platforms for the development of new LBPs according to a strategic workflow (Figure 4). A collection of microorganisms of interest must be tested by conventional *in vitro* methods to select strains that meet the eligibility criteria defined in the literature. These candidates are then characterized using *C. elegans* and preclinical models. The final necessary step before an application in human health remains the clinical trials. Future *C. elegans* research objectives are to characterize the mode of action of probiotic strains to better understand their effects on humans and to develop even better live biotherapeutic products to treat a larger panel of human dysbiosis-related affections. Even though this nematode has become one of the most intensively studied models in biological research, a full analysis of its biology would warrant further integration of this information into the research approaches (171). In addition to the strain-specific approaches, we must also take into consideration the industrial process for a large-scale production of a probiotic strain. A native probiotic strain may present different or potentiated properties depending on the culture conditions, the drying process and the galenic (153, 156, 172, 173). *C. elegans* could be a strategic model to test the secondary effects of new drugs (impact on its lifespan) or new formulations.

Studies using *C. elegans* as an *in vivo* model with the aim to characterize probiotic potential of bacterial strains are largely focused on lactic acid bacteria. Some other kinds of microorganisms such as anaerobic bacteria and fungi could be of interest as probiotics and their characteristics could be evaluated using *C. elegans*. Few studies have been published concerning these kinds of microorganisms (*Aeromonas, Vibrio cholera, Mycobacterium avium*). Because no published data exist concerning the physicochemical environment in *C. elegans* intestine (pH, oxygen level), the feasibility of anaerobic bacteria studies on *C. elegans* must be investigated.

A collection of human pathogens has been studied using *C. elegans* model (Table 2). Future opportunities could be to enlarge the panel of pathogens studied. The study of Hwang et al. (174) demonstrated that *C*. *elegans* is an effective model to examine and compare the pathogenicity and virulence variation of STEC strains (24 strains tested) to that of *E*. *coli* OP50 strain. White and Herman (80) demonstrated the merit of a *C. elegans* approach in the identification of novel genes that are involved in combating the emerging nosocomial bacterial pathogen *Stenotrophomonas maltophilia* infection. The work of Wan et al. (175) provides new insights into the pathogenesis of *B. thuringiensis*, and highlights the importance of breaking down host epithelial junctions for a successful infection. A similar mechanism could be used by other pathogen-host interactions since epithelial junctions are conserved structures from insects to mammals. Efforts have to be made to further elucidate the evolutionary conserved mechanisms of bacterial and fungal pathogenesis as well as of host-microbes interactions. A lethal impact of microbiota of inflamed colon from inflammatory bowel disease (IBD) patient's toxicity in *C. elegans* compared with microbiota from the non-inflamed site of the colon was reported. Moreover, in these conditions, the co-exposure of human pathogen *Giardia duodenalis* increased lethal toxicity (176). Another study showed that co-infection of *C. elegans* with two human pathogens such as *C. albicans* and *E. faecalis*, had less severe toxic impact than either pathogen alone. The authors hypothesized that this observed effect was due to inhibition of *C. albicans* induced hyphal morphogenesis by *E. faecalis* derived antifungal compounds (177). This worm model offers the opportunity to explore potential trans-kingdom interactions, to define the function of the commensal microbiota and the host-microbiota-pathogens interactions. The next challenge is to use *C. elegans* to study multi-infections to define the relationships and the influences of microbes on each other. In the coming years, this *in vivo* model could be widely used to better characterize microorganisms using omics approaches associated with automation. Although being one of the most common procedures required during *C. elegans* research, counting nematode numbers is a time-consuming and fastidious process, and prone to errors. A new estimation procedure needs to be developed to make faster and reliable data collection from a worm population. A technical progress through automation would allow to enlarge applications to the screening of large microorganisms collections (178, 179). Microfluidic represents new perspectives to manipulate *C. elegans* in a high-throughput fashion to perform automated studies using high-resolution imaging methods (180). There is an urgent need for the discovery of effective new antimicrobial agents to combat the rise of bacterial drug resistance. High-throughput screening (HTS) in whole-animal infection models could represent a powerful tool for identifying compounds that show antibacterial activity and low host toxicity (181–183). Research works are focused on LAB and their anti-pathogen activities, their capacities to extend lifespan of *C. elegans* and to manage with oxidative stress and inflammation, but some domain of research in relation with human health and nutrition have not been exploited yet.

**Table 2:** Pathogen—LAB interactions studied in the *C. elegans* intestine.

**Kingdoms**	**Pathogens**	**LABs**	**Mechanisms**	**References**
Bacteria	*S. enterica*	*L. helveticus* *L. plantarum* *L. rhamnosus* *B. infantis* *B. longum* *E. faecium*	Immunotolerance Not described Not described	(84) (132) (35)
	*P. aeruginosa*	*L. brevis* *P. acidilatici* *P. pentosaceus*	Not described	(32)
	*S. aureus*	*L. plantarum* *L. zeae* *L. casei* *L. rhamnosus* *L. acidophilus*	Not described TIR-1, PMK-1 and BAR-1 pathways	(74) (160)
	*E. coli* (clinical isolates)	*L. plantarum* *L. paracasei*	Not described Not described	(154) (89)
	ETEC	*L. zeae* *L. casei* *L. plantarum* *L. rhamnosus*	Inhibition of *E. coli* virulence Not described	(155) (36)
	EHEC	*E. faecalis* *L. acidophilus*	Inhibition of virulence Inhibition of virulence	(156) (157)
	*L. pneumophila*	*B. infantis*	p38 MAPK pathway	(159)
	*E. faecalis*	*L. acidophilus*	TIR-1, PMK-1 and BAR-1 pathways	(160)
Fungi	*C. albicans*	*L. rhamnosus* *L. paracasei*	DAF-16 and p38 MAPK pathway Filamentation inhibition	(90, 150) (151)

According to its natural complex living environment, it is likely that the wild *C. elegans* intestine is colonized with many different microorganisms. With the aim to be ever closer to the human intestine, one of the future challenges will be to deal with a diversified worm fed, to manage colonization of *C. elegans* intestine with a consortium of strains (184). It would be of interest to enlarge the panel of experiments with *C. elegans*, feeding it with a complex microbiota (intestinal, vaginal, buccal, etc.) or with fermented food (raw milk cheese) to evaluate the impact of these microorganisms mixes on its lifespan, to observe the transit of the microorganisms through the intestine and then their potential to colonize it. The feasibility of using this model to generate human-like microbiota in the worm intestine has never been reported but represents a scientific challenge for large scales studies focusing on host-microbiota or host-holobiont.

## Conclusion

The literature mainly highlights the probiotic impact of LAB strains exerting highly beneficial effects on *C. elegans* whether it be on biotic and abiotic stresses or on immunity. The fact that the worm presents genetic, functional, and physiological similarities with humans represents a plus value to *in vitro* cell-culture strategies to investigate molecular probiotic mechanisms. The *C. elegans* model provides a large range of opportunities to explore trans-kingdom interactions and to solve questioning about the molecular aspect of these interactions. It represents a pertinent, easy-to-handle, cheap and rapid tool for the screening of microorganisms' collections looking for specific probiotic characters. *C. elegans* should emerge as one of the most powerful, bioethical, and pertinent go-between model host between *in vitro* and mammalian models.

## Author Contributions

CP, CC, and SB writing-original draft. AN and SB supervision. All authors contributed to the article and approved the submitted version.

## Conflict of Interest

AN had an institutional affiliation with the company which manufactures Lcr35 products. AN was employed by the company biose^®^ Industrie. The remaining authors declare that the research was conducted in the absence of any commercial or financial relationships that could be construed as a potential conflict of interest.
